# Retinoic acid receptor gamma impacts cellular adhesion, Alpha5Beta1 integrin expression and proliferation in K562 cells

**DOI:** 10.1371/journal.pone.0178116

**Published:** 2017-05-26

**Authors:** Melissa D. Kelley, Raynin Phomakay, Madison Lee, Victoria Niedzwiedz, Rachel Mayo

**Affiliations:** Department of Chemistry and Biochemistry, University of Central Arkansas, Conway, Arkansas, United States of America; Thomas Jefferson University, UNITED STATES

## Abstract

The interplay between cellular adhesion and proliferation is complex; however, integrins, particularly the α5β1 subset, play a pivotal role in orchestrating critical cellular signals that culminate in cellular adhesion and growth. Retinoids modify the expression of a variety of adhesive/proliferative signaling proteins including α5β1 integrins; however, the role of specific retinoic acid receptors involved in these processes has not been elucidated. In this study, the effect of all-*trans*-retinoic acid receptor (RAR) agonists on K562 cellular adhesion, proliferation, and α5β1 integrin cell surface expression was investigated. RARγ agonist exposure increased K562 cellular adhesion to RGD containing extracellular matrix proteins fibronectin and FN-120 in a time- and concentration dependent manner, while RARα or RARβ agonist treatment had no effect on cellular adhesion. Due to the novel RARγ- dependent cellular adhesion response exhibited by K562 cells, we examined α5 and β1 integrin subunit expression when K562 cells were exposed to retinoid agonists or vehicle for 24, 48, 72 or 96 hours. Our data demonstrates no differences in K562 cell surface expression of the α5 integrin subunit when cells were exposed to RARα, RARβ, or RARγ agonists for all time points tested. In contrast, RARγ agonist exposure resulted in an increase in cell surface β1 integrin subunit expression within 48 hours that was sustained at 72 and 96 hours. Finally, we demonstrate that while exposure to RARα or RARβ agonists have no effect on K562 cellular proliferation, the RARγ agonist significantly dampens K562 cellular proliferation levels in a time- and concentration- dependent manner. Our study is the first to report that treatment with a RARγ specific agonist augments cellular adhesion to α5β1 integrin substrates, increases cell surface levels of the β1 integrin subunit, and dampens cellular proliferation in a time and concentration dependent manner in a human erythroleukemia cell line.

## Introduction

Retinoids, vitamin A and its analogs, play a critical role in modulating the precise balance between cellular adhesion and proliferation that is required for maintenance of proper cell homeostasis [1–7]. Biologically active metabolites of vitamin A, all-*trans*-retinoic acid and 9-*cis*-retinoic acid are reported to mediate a number of cellular functions including differentiation, proliferation, adhesion, transmigration and more recently integrin expression [8–10].

All-*trans*-retinoic acid (*t*-RA) and 9-*cis*-retinoic acid (9-*cis*-RA) activate retinoic acid receptors (RAR α, β, and γ) and retinoid X receptors (RXR α, β, and γ). These retinoids act as ligand-dependent transcription factors with *t*-RA activating RARs, while 9-*cis*-RA serves as a pan-agonist for RARs and RXRs [11–15]. These receptor complexes act as heterodimers or homodimers binding to specific retinoid response elements, RARE and RXRE, in the promoter of target genes [16]. These distinct retinoid receptor partnerships differentially regulate transcription of a number of targets who actively participate in the complex interplay of cellular adhesion and proliferation, including integrins, and their extracellular matrix counter-receptors [9, 17–20].

Integrin-mediated adhesion to the extracellular matrix stringently regulates cell cycle progression [21, 22]. Integrins, a family of transmembrane heterodimeric receptors consisting of non-covalently linked α and β subunits are the principle receptors involved in attachment to the extracellular matrix. The α5β1 integrins are fibronectin (FN) receptors with the RGD sequence in the FNIII domain 10 being the crucial attachment site for α5β1 [23]. Signaling processes that are crucial for regulating cell proliferation follows initial α5β1 integrin binding of the RGD sites in FN [24–28]. There have been a number of studies specifically demonstrating that altered α5β1 integrin expression results in modulated cellular proliferation [29–32]. Interestingly, retinoids have been implicated in altering integrins, including α5β1 [18, 20].

Although the ability of *t*-RA and 9-*cis*-RA to alter cell adhesion and proliferation is addressed in the literature, there is a shortage of information regarding which retinoid receptors govern these particular cellular functions through modulation of the α5β1 integrin subset. We present evidence that exposure to a RARγ agonist augments adhesion to both fibronectin and the α5β1 specific chymotryptic fragment of fibronectin (FN-120) in a time and concentration dependent manner. Further, we investigated the effects of retinoid agonists on both the integrin α5 and β1 subunit cell surface expression in K562 cells and report that the β1integrin subunit is increased on the cell surface in response to RARγ agonist treatment. Finally, we demonstrate that the RARγ agonist dampens cellular proliferation in a time and concentration dependent manner. Our study is the first to report that a RARγ agonist augments cellular adhesion, dampens cellular proliferation, and increases β1 integrin cell surface expression in a human erythroleukemia cell line.

## Materials and methods

### Reagents and chemicals

Human fibronectin (FN) was purchased from BD Biosciences (Bedford, MA). The purified human fibronectin alpha-chymotryptic fragment, FN-120, was purchased from Millipore (Temecula, CA). Anti-integrin α5 FITC conjugated antibody (SAM-1), and MOPC-21 (isotype control) was purchased from Abcam (Cambridge, MA). The anti-integrin β1 FITC conjugated antibody was purchased from Millipore. Retinoid agonists RARα (AM 580) RARβ (AC 55649) and RARγ (CD 437) were purchased from Tocris (Minneapolis, MN). Agonists were stored at -20°C or +4°C. Retinoids were dissolved at the desired concentration in ethanol. The compound *p*-nitro-phenyl phosphate was purchased from Sigma (St. Louis, MO).

### Cell culture

The human cell line K562 was obtained from ATCC (Manassas, VA) and maintained in RPMI 1640 supplemented with 10 mM HEPES, 1mM sodium pyruvate, 10% (v/v) fetal bovine serum, 1% L-glutamine and 1% penicillin-streptomycin at 37°C in an atmosphere of 5% CO_2_.

### Cellular adhesion assays

Static adhesion assays to immobilized ligands were adapted from established techniques [33, 34]. Briefly, fibronectin, or FN-120 were immobilized at desired concentrations on 96-well Immulon-2 HB microtiter plates (Thermo Scientific, Waltham, MA) in a total volume of 100 μl of 0.1 M NaHCO_3_ pH 8.4 overnight at 4°C. Nonspecific adhesion was minimized by blocking wells with 2% (w/v) bovine serum albumin (BSA) in 0.1 M NaHCO_3_ at room temperature for 1 hr. K562 cells were cultured for the designated times (24, 48, 72, or 96 hrs) in the presence of RARα agonist (AM 580) (1 μM), RARβ agonist (AC 55649) (1 μM), RARγ agonist (CD 437) (0.125–1 μM), or an equimolar concentration of ethanol. Before addition to wells, cells were washed twice in HEPES-Tyrodes buffer, enumerated, and added to wells (9x10^4^ cells/well) in HEPES-Tyrodes with 1 mM MnCl_2_. Cells were incubated in the 30 min (fibronectin ligand) or 1 hr (FN-120 ligand) at 37°C in 5% CO_2_. After three consecutive washes with HEPES-Tyrodes, wells were analyzed for bound cells by determining the relative cellular acid phosphatase activity within each well. Phosphatase assay buffer (1% v/v Triton X-100, 50 mM sodium acetate at pH 5.0 and 6 mg/mL *p*-nitrophenyl phosphate) was added to wells, and wells were incubated with the substrate for 30 minutes at 37°C. Color was disclosed by addition of 50 μl/well of 1 *N* NaOH. Absorbance values were obtained at 405 nm using a Biotek microplate reader. Adhesion values obtained with wells coated exclusively with BSA were considered as background values for each experimental condition and were subtracted before reporting final values. Adhesion values were normalized using Percent Vehicle = (Abs_(treatment)_/Abs_(vehicle)_) X 100. Results are shown as means ± SD, n = 6.

### Flow cytometry

Cells were treated with 1 μM RARα agonist (AM 580), RARβ agonist (AC 55649), RARγ agonist (CD 437), or an equimolar concentration of ethanol for designated time periods (24–96 hrs). Cells were harvested and washed twice in PBS containing 3.0% (w/v) BSA (PBS-BSA). 1.0 x 10^6^ cells were re-suspended in a total volume of 1 mL PBS-BSA, anti-α5 integrin antibody, anti-β1-integrin antibody, or isotype control (10 μg/mL) was added, and cells were incubated on ice for 30 min. Unbound antibody was removed by three final washes in PBS-BSA, and fluorescent intensity was analyzed on Beckman-Coulter Quanta SC flow cytometer (Beckman-Coulter, Inc. Brea, CA).

### BrdU cell proliferation assay

The ELISA-based BrdU cell proliferation kit was purchased from Chemicon International (Temecula, CA). Cells were plated at 2.5 x 10^4^ cells/mL in 100 μl culture media containing RARα agonist (AM 580) (1 μM), RARβ agonist (AC 55649) (1 μM), RARγ agonist (CD 437) (0.125–1 μM), or an equimolar concentration of ethanol. K562 cells were cultured for a total of 24–96 hrs at 37°C at 5% CO_2_ atmosphere. All treatments were diluted to their final concentrations with media to desired concentrations to avoid proliferation effects due to high ethanol concentrations. Absorbance values were obtained with a microplate reader at 450 nm. BrdU incorporation was assessed by ELISA and proliferation values were normalized using Percent Vehicle = (Abs_(treatment)_/Abs_(vehicle)_) X 100. Results are shown as means ± SD, n = 6.

### Statistical analysis

LSD was used to test for differences among groups (P = 0.01)

## Results

### RARγ agonist exposure augments K562 cellular adhesion to RGD containing extracellular matrix proteins in a time-dependent manner

**Fig 1:**
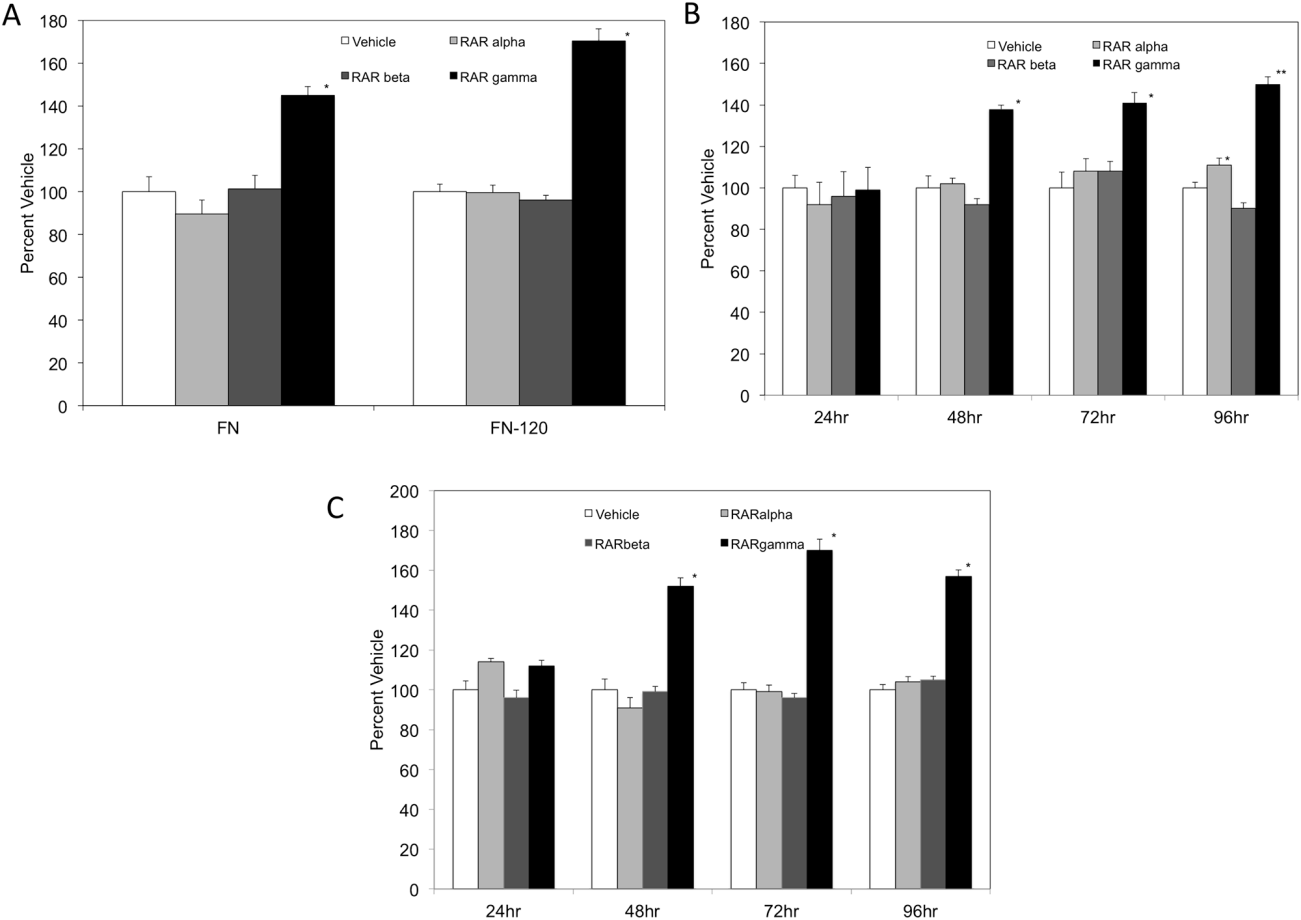
K562 cellular adhesion to fibronectin and FN-120 is increased in a time-dependent manner in the presence of an RARγ agonist. (A) Microtiter wells were coated with 5 μg/mL fibronectin or FN-120. K562 cells cultured for 72 hrs in the presence of vehicle (*white bars*), 1 μM RARα agonist (*light gray bars*), RARβ agonist (*dark gray bars*) or RARγ agonist (*black bars*) were added to wells (9×10^4^ cells/well) in Hepes-Tyrodes buffer containing 1 mM MnCl_2_. (B) K562 cells cultured for 24, 48, 72, or 96 hrs in the presence of vehicle (*white bars*), 1 μM RARα agonist (*light gray bars*), RARβ agonist (*dark gray bars*) or RARγ agonist (*black bars*) were added to wells coated with 5 μg/mL fibronectin, and adhesion assays were repeated. C) K562 cells cultured for 24, 48, 72, or 96 hrs with vehicle (*white bars*), 1 μM RARα agonist (*light gray bars*), RARβ agonist (*dark gray bars*) or RARγ agonist (*black bars*) were added to wells coated with 5 μg/mL FN-120, and adhesion assays were repeated. Percent Vehicle = Abs_405nm(treatment)_/Abs_405nm(vehicle)_ X 100. Results are expressed as means ± SD, n = 6, LSD was used to test for differences among groups. Means followed by * or ** are significantly different (P = 0.01).

Retinoids have been established as having a role in governing integrin-dependent cellular adhesion [18, 35]. In K562 cells, retinoids have been demonstrated to restore cellular adhesion to RGD substrates [18]. However, there is paucity amount of information regarding which retinoid receptors govern cellular adhesion, particularly in K562 cells. To evaluate if retinoid receptor agonists alters cellular adhesion in K562 cells, we employed a static cell adhesion assay utilizing fibronectin (FN) or FN-120, which were selected due to their well-defined RGD binding sites. As shown in Fig 1A, K562 cells treated with 1 μM RARα or RARβ agonists for 72 hours had similar levels of cellular adhesion on fibronectin at 5 μg/mL as compared to vehicle. Conversely, RARγ agonist treated cells had a 44% increase in adhesion compared to vehicle treatment on the fibronectin ligand. Fibronectin contains binding sites for a number of integrins, including the α5β1 subset. To determine the contribution to cellular adhesion by only the α5β1 integrin subset, the 120-kDa chymotryptic fragment with the RGD recognition sequence (FN-120) was utilized and cellular adhesion assays were repeated (Fig 1A). RARα and RARβ agonists treated cells had cellular adhesion values comparable to the vehicle. Interestingly, on the FN-120 substrate, RARγ agonist treated cells had a 70% increase in cellular adhesion when compared vehicle treated cells.

To address if the observed augmented adhesion was time-dependent, we restricted the exposure of K562 cells to these agonists for 24, 48, 72 or 96 hrs. When cells were added to wells coated with FN, similar levels of cellular adhesion were obtained for all treatment groups cultured for 24 hours (Fig 1B). At 48 hours, a significant increase in cellular adhesion was observed in K562 cells treated with the RARγ agonist on the FN ligand. However, there were no statistical differences between cellular adhesion levels of K562 cells treated with vehicle, RARα agonist, or RARβ agonists for 48 hours. At 72 hours, K562 cells exposed to the RARγ agonist obtained statistically higher cellular adhesion levels with an increase of 41% in cellular adhesion compared to vehicle treatment only on the FN substrate. K562 cells cultured with RARα or RARβ agonists for 72 hours had comparable levels of cellular adhesion to vehicle treatment alone. At 96 hours, significant levels of cellular adhesion were observed with cells dosed with the RARγ agonist with a 50% increase in cellular adhesion compared to vehicle control.

As shown in Fig 1C, a similar trend in cellular adhesion was observed when cells were added to wells coated with FN-120. K562 cells cultured in the presence of retinoid receptor agonists for 24 hours had no statistical differences between cellular adhesion levels as compared to vehicle control. At 48 hours, a significant increase in cellular adhesion was observed when cells were treated with the RARγ agonist utilizing the FN-120 ligand. There were no statistical differences between cellular adhesion levels of K562 cells treated with vehicle, RARα agonist, or RARβ agonists for 48 hours. At 72 hours, K562 cells exposed to the RARγ agonist had a robust 70% increase in cellular adhesion compared to vehicle treatment only. Cells dosed with RARα or RARβ agonists had comparable levels of cellular adhesion to vehicle treatment alone. At 96 hours, a significant increase in cellular adhesion (57%) was observed when cells were dosed with the RARγ agonist compared to vehicle control. However, there were no statistical differences between cellular adhesion levels of K562 cells treated with vehicle, RARα agonist, or the RARβ agonist at 96 hours. Collectively, our data demonstrates that treatment with the RARγ agonist results in a robust increase in cellular adhesion that is time dependent with significant augmented cellular adhesion observed within 48 hours of RARγ agonist treatment.

### RARγ agonist increases K562 cellular adhesion to FN and FN-120 in a concentration-dependent manner

**Fig 2:**
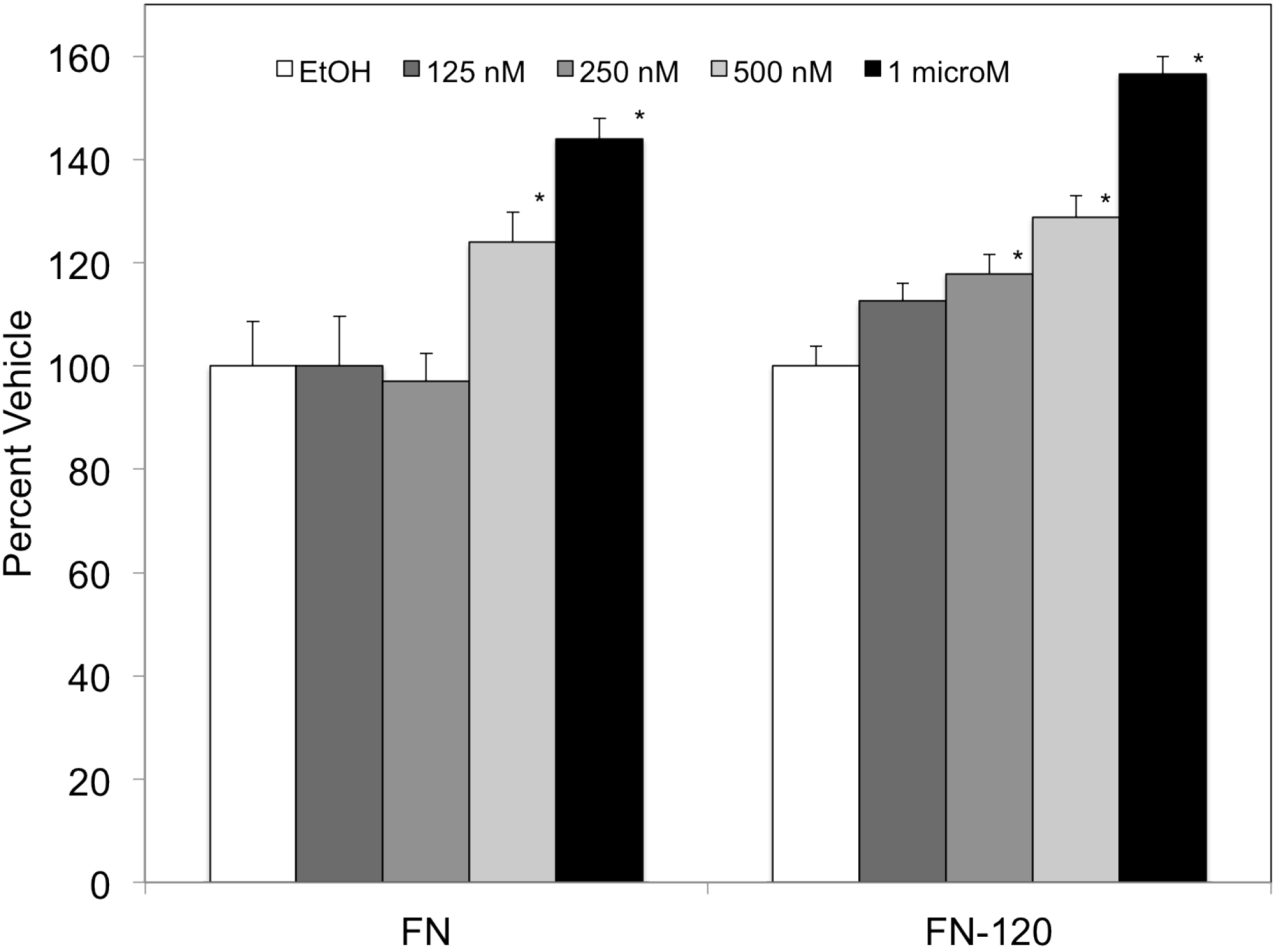
RARγ agonist exposure augments K562 cellular adhesion to RGD containing extracellular matrix proteins in a concentration dependent manner. (A) K562 cells were cultured for 72 hrs in the presence of vehicle (*white bars*), 125 nM RARγ agonist (*dark gray bars)*, 250 nM RARγ agonist (*medium gray bars)*, 500 nM RARγ agonist (*light gray bars)* or 1 μM RARγ agonist (*black bars)* After 72 hrs of treatment, K562 cells were added to wells (9×10^4^ cells/well) in Hepes-Tyrodes buffer containing 1 mM MnCl_2_ to wells coated with 5 μg/mL of fibronectin, or FN-120. Percent Vehicle = Abs_405nm(treatment)_/Abs_405nm(vehicle)_ X 100. Results are expressed as means ± SD, n = 6, LSD was used to test for differences among groups. Means followed by * are significantly different (P = 0.01).

Retinoids have been evaluated for their therapeutic potential; however, their use has been limited due to toxicity and teratogenic side effects [1]. Since the RARγ agonist increases cellular adhesion to both fibronectin and FN-120 at 1μM, we evaluated whether lower concentrations of the RAR gamma agonist confers cellular adhesion to these ligands. The agonist concentration chosen were based on reported EC_50_ values [36]. As shown in Fig 2, K562 cells cultured in the presence of the RARγ agonist had increased cellular adhesion to FN starting at a concentration of 500 nM. When cells were dosed with RARγ agonist at 500 nM or 1 μM, cellular adhesion was increased by 24% and 44%, respectively. Cellular adhesion values were comparable to the vehicle when K562 cells were dosed with 125 nM or 250 nM RARγ agonist. Interestingly, when the FN-120 ligand was utilized a statistically significant increase in cellular adhesion was observed at the lowest RARγ agonist concentration of 125 nM. As shown in Fig 2, K562 cells cultured in the presence of 125 nM, 250 nM, 500nM or 1 μM RARγ agonist had continual increasing levels of adhesion to FN-120 ligand.

### RARγ agonist augments cell surface expression of the β1 integrin subunit in a time-dependent manner

**Fig 3:**
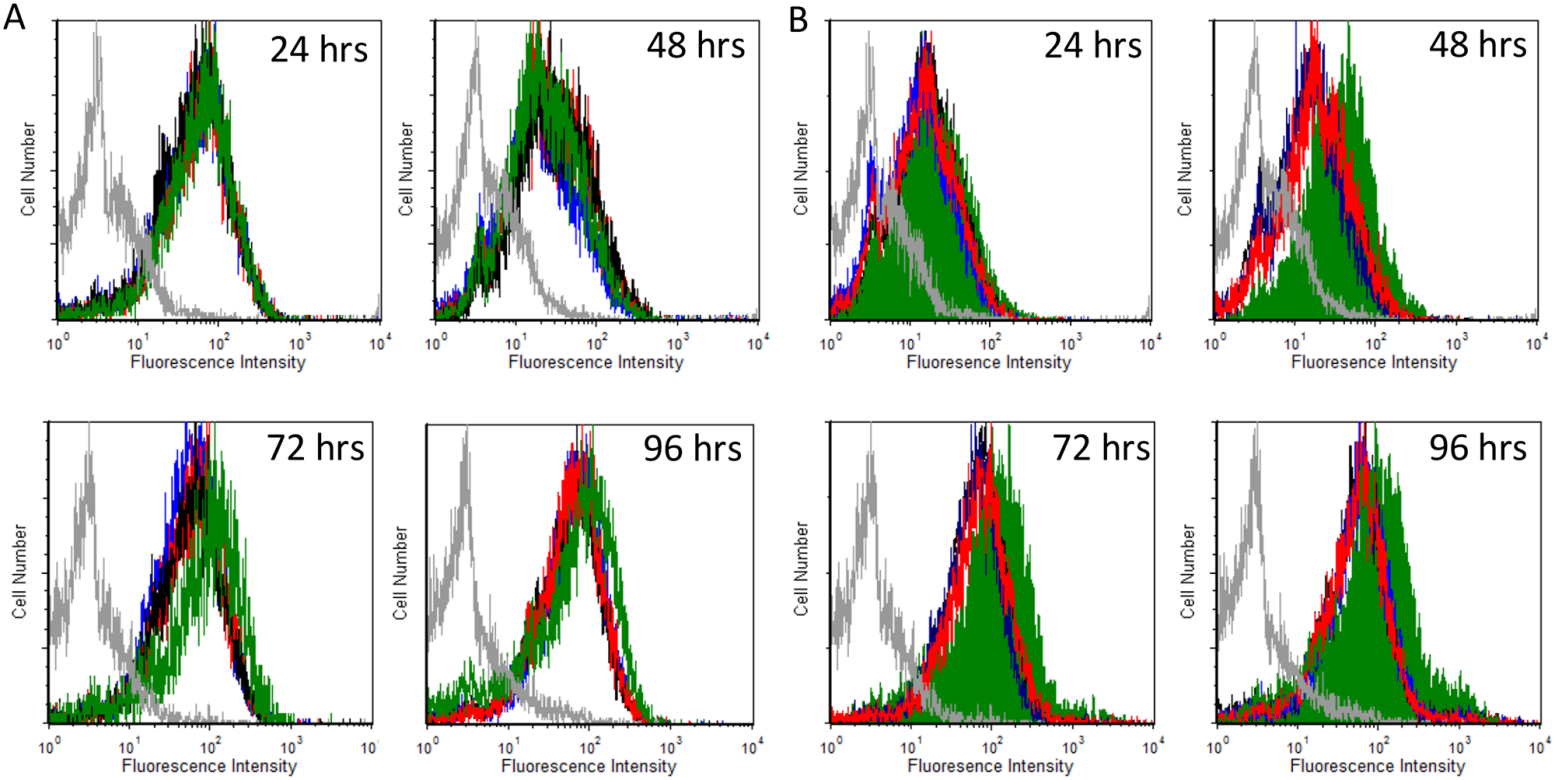
An RAR agonist augments the cell surface expression of the β1 integrin subunit in a time-dependent manner. (A) The extent of mAb SAM-1 (anti-α5) binding to K562 cells exposed to vehicle, 1 μM RARα agonist, RARβ agonist, or RARγ agonist for 24, 48, 72, or 96 hrs was assessed by FACS analysis. SAM-1 was used at a concentration of 10 μg/mL. Cell surface levels of the αα5 integrin subunit obtained with vehicle cells (*black histogram*), RAR alpha agonist (*blue histogram*), RAR beta agonist (*red histogram*), RAR gamma agonist (*green histogram*), or an antibody isotype control (*gray histogram*) are shown. (B) The extent of mAb MB 1.2 (anti-β1) binding to K562 cells exposed to vehicle, 1 μM RARα agonist, RARβ agonist, or RARγ agonist for 24, 48, 72, or 96 hrs was assessed by FACS analysis. MB 1.2 (anti-β1) mAb was used at a concentration of 10 μg/mL. Cell surface levels of the β1 integrin subunit obtained with vehicle cells (*black histogram*), RAR alpha agonist (*blue histogram*), RAR beta agonist (*red histogram*), RAR gamma agonist (*green filled histogram*), or an antibody isotype control (*gray histogram*) are shown.

Retinoids have been shown to modulate the integrin repertoire expressed on the cell surface of a number of cell lines, including K562 cells [17, 18, 35]. A number of integrin subunits have been profiled on the K562 cell surface in the presence of 9-*cis*-RA and recent studies have shown that retinoids are capable of impacting only the α5β1 subset on K562 cells [18]. Due to the novel adhesion response exhibited by K562 cells, we examined integrin expression of α5 and β1 subunits when K562 cells were exposed to 1 μM RARα, β, or γ agonists or equimolar concentration of vehicle for 24, 48, 72 or 96 hours. Utilizing the anti-α5 integrin antibody, our data demonstrates no differences in the α5 integrin subunit expression on the cell surface when K562 cells were exposed to either RARα, RARβ, or RARγ agonists for 24, 48, 72, or 96 hours (Fig 3A). However in contrast to the cell surface α5 integrin subunit findings, K562 cells exposed to the RARγ agonist had an increase in cell surface β1 integrin subunit expression compared to the vehicle within 48 hours (Fig 3B). Interestingly, K562 cell cultured with the RARγ agonist demonstrate an increase in the cell surface β1 integrin expression at 72 hours when compared to vehicle. At 96 hours, RARγ agonist treated cells have an increase in cell surface β1 integrin subunit levels compared to vehicle. Cell surface levels of the β1 integrin subunit is comparable to the vehicle when K562 cells are exposed to the RARα or RARβ agonists for 48, 72 or 96 hours. Our results shown in Fig 3B add support to the cellular adhesion assay findings shown in Fig 1A–1C. Collectively, our data demonstrate that the RARγ agonist promotes cellular adhesion to α5β1 integrin ligands through augmenting cell surface β1 integrin expression.

### RARγ agonist exposure dampens K562 cellular proliferation in a time-and concentration-dependent manner

**Fig 4:**
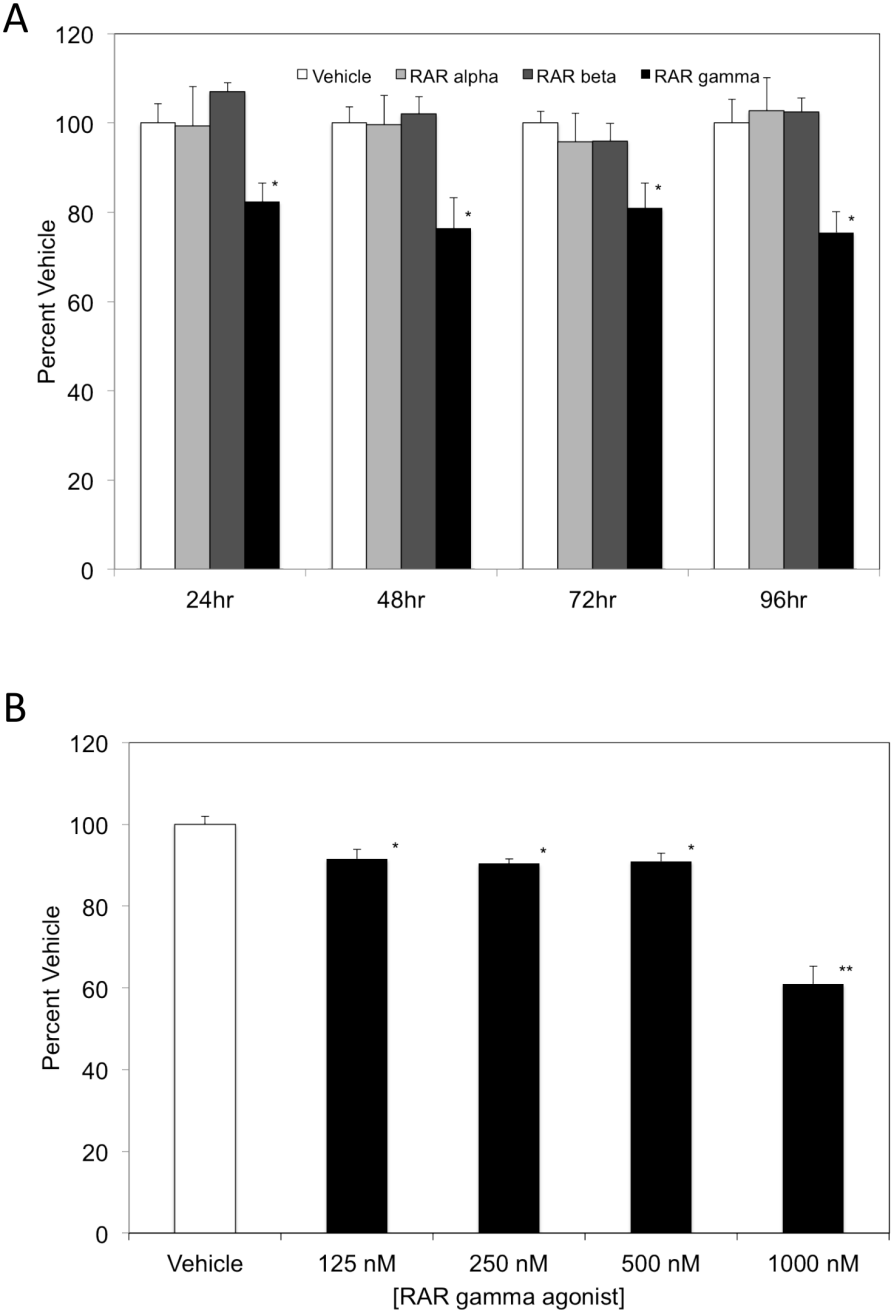
K562 cellular proliferation is decreased by RARγ agonist exposure in a time-and concentration-dependent manner. **(A)** K562 cells cultured for 72 hrs in the presence of vehicle (*white bars*), 1 μM RARα agonist (*light gray bars*), RARβ agonist (*dark gray bars*) or RARγ agonist (*black bars*), respectively, for 24, 48, 72 or 96 hrs. BrdU incorporation was assessed by ELISA and proliferation values were normalized using Percent Vehicle = (Abs_(treatment)_/Abs_(vehicle)_) X 100. Results are shown as means × SD, n = 12, LSD was used to test for differences among groups. Means followed by * are significantly different (P = 0.01). (B) K562 cells were cultured for 72 hrs in the presence of vehicle (*white bars*) or RARγ agonist at varying concentrations (*black bars*). BrdU incorporation was assessed by ELISA and proliferation values were normalized using Percent Vehicle = (Abs_(treatment)_/Abs_(vehicle)_) X 100. Results are shown as means ± SD, n = 6, LSD was used to test for differences among groups. Means followed by * or ** are significantly different (P = 0.01).

The balance between cellular adhesion and proliferation is fundamental to maintaining cell homeostasis with cells requiring anchorage to extracellular matrix proteins to proliferate. Interestingly, there have been studies demonstrating that unoccupied α5β1 integrin causes cell growth inhibition [31, 32, 37]. Additionally, studies have demonstrated that over-expression of the α5β1 integrin results in suppressed cellular proliferation [29–32]. Due to the novel cellular adhesion response elicited by the RARγ agonist and the corresponding increase in the integrin β1 cell surface expression upon RARγ agonist exposure, we assessed the impact of this agonist on K562 cellular proliferation. K562 cells were cultured in the presence of vehicle, RARα agonist, RARβ agonist, or RARγ agonist for 24, 48, 72, or 96 hours and proliferation levels were assessed as shown in Fig 4A. We observe a 18% decrease in proliferation levels when cells were treated with the RARγ agonist, while cells cultured with either the RARα or RARβ agonist had comparable proliferation levels to the vehicle at 24 hrs. At 48 hours, proliferation levels were decreased by 24% in the presence of the RARγ agonist when compared to the vehicle. Interestingly, a similar trend was noted at 72 and 96 hours with a decrease in proliferation levels of 19% and 25%, respectively when K562 cells were treated with the RARγ agonist. At 48, 72 and 96 hours, cells treated with either the RARα or RARβ agonists had comparable proliferation levels to the vehicle.

It is well established that retinoids are modulators of proliferation. A number of studies have focused on the role of specific RARs in regulating proliferation with a limited number of investigations focusing on the RARγ agonist at high concentrations [36, 38–40]. Due to the novel adhesion response demonstrated at lower concentrations of the RARγ agonist, we assessed whether the same RARγ agonist concentrations impact cellular proliferation. K562 cells were cultured in the presence of the RARγ agonist for 72 hours at concentrations ranging from 125 nM-1μM. As shown in Fig 4B, we observe a slight, but significant decrease in proliferation compared to vehicle when cells were cultured with the RARγ agonist at the lowest dose of 125 nM. A similar trend was observed when cells were treated with either the 250 nM or 500 nM RARγ agonist with a decrease in cellular proliferation of approximately 10% compared to vehicle control. Interestingly, we observe a significant and large decrease in cellular proliferation when cells are dosed at the higher concentration of 1 μM of the RARγ agonist. Collectively, our data demonstrate a significant decrease in cellular proliferation when K562 cells are exposed to the RARγ agonist that is time and concentration dependent.

## Discussion

The requirement of vitamin A in maintaining cell homeostasis, particularly in regards to immunity has been appreciated since the earliest studies in the 1920’s [41]. However, the molecular mechanisms by which retinoids elicit their effects was not illuminated until the discovery of retinoid receptors [11–16]. The current study focuses on the effects of RAR specific agonists on cellular adhesion and proliferation in the human erythroleukemia cell line, K562. We present evidence that the RARγ agonist augments cellular adhesion to RGD containing extracellular matrix proteins fibronectin and FN-120, which contain the binding sites for α5β1 integrins in a time-and concentration-dependent manner. Recently, K562 cells have been demonstrated to be retinoid response in the presence of troglitazone, a PPARγ ligand [18]. In the presence of troglitazone, K562 cellular adhesion is dampened; however, upon the addition of the RAR/RXR pan-agonist, 9-*cis*-RA, cellular adhesion is restored to levels comparable vehicle treatment alone. Further, 9-*cis*-RA increases cell surface levels of the integrin subunit α5 when cells were exposed to troglitazone [18], while cell surface integrin subunit β1 levels remain unchanged. Our current work may provide insight into how retinoids modulate both alpha 5 and beta 1 integrin subunit expression. Since integrins function as heterodimeric partners, we suggest that RARγ retinoid receptor is responsible for the up-regulation of the beta 1 subunit, while the alpha 5 subunit may be transcriptionally regulated by the pan-agonist 9-*cis*-RA. It is plausible to suggest that through differential RAR/RXR pairings or promiscuous RXR partnerships different integrin subsets may be expressed to modulate cellular functions including cellular adhesion and proliferation, which is required for proper cell homeostasis. Additionally, the RARγ receptor isotype has been shown to differentially recruit specific components of the transcriptional machinery [42]. Both of these mechanisms may be involved in the augmented cellular adhesion observed in the presence of the RARγ agonist.

All-*trans*-retinoic acid and 9-*cis*-retinoic acid have been implicated in modulating integrin-dependent and -independent cellular adhesion [18, 33, 35, 43]. Interestingly, we observe a slight but significant increase in cellular adhesion when cells were exposed to the RARα agonist for 96 hours on the fibronectin ligand (Fig 1B). However, we do not observe an increase in cellular adhesion on the α5β1 integrin specific FN-120 ligand in the presence of the RARα agonist (Fig 1C). Further, we do not detect an observable change in the cellular surface levels of the alpha 5 or beta 1 integrin subunit expression (Fig 3A and 3B) in the presence of the RAR α agonist. Fibronectin contains binding sites for α5β1, αvβ3 and α4β7 integrins. Yet, K562 cells have not been reported to contain the α4β7 integrin [35]. In a recent study, a number of integrins were profiled in K562 cells exposed to 9-*cis*-RA [18]. This study demonstrated that 9-*cis*-RA exposure did not increase α8, β3, αvβ5, or αvβ3 integrins on the cell surface of K562 cells and this data reflects very low surface levels regardless of vehicle or 9-*cis*-RA treatment group. Recently, the RARα specific isotype has been shown to induce integrin-independent cellular adhesion in RPMI 8866 cells [43]. It is plausible to suggest K562 cells exposed to the RARα agonist for 96 hours may have acquired integrin-independent adhesion strategies that are dependent on classical retinoid induced genetic modifications. Given the promiscuous nature of retinoid receptors and the complex signal transduction pathways involved in integrin-dependent and -independent cellular adhesion and migration, it is likely that distinct retinoid receptor pairings may facilitate a number of cellular events that modulate cellular adhesion and proliferation.

Our data reflect that the RARγ isotype augments cellular adhesion and β1 integrin cell surface expression, while dampening cellular proliferation. It is interesting to note that there appears to be a reciprocal relationship between cellular adhesion and proliferation that is mediated through the RARγ isotype. Within the past three decades, the mechanisms by which retinoids elicit their classical genomic effects have been clearly established. There is a commonality between RAR isotypes as evidence by their ability to modulate transcription of common target genes. However, there is a disparity in retinoid receptor isotype functions [40]. For example, the RARγ receptor isotype has been shown to differentially recruit specific components of the transcriptional machinery [42]. Additionally, there has been evidence of antagonism between RAR isotypes with RARγ inhibiting functions of other RAR isotypes [40]. Moreover, different retinoid receptor isotypes have different transcriptional efficiency for the same target gene [40]. Further, others have demonstrated that low doses of selective RAR agonists acting in concert with their heterodimeric RXR partner may have more restricted effects on *t*-RA dependent transcription of target genes, thus avoiding the large number of adverse pleiotropic effects of *t*-RA [44]. Clearly, there is paucity of information regarding how RAR isotypes function to modulate the complex interplay between cellular adhesion and proliferation; however, determining the role that the RARγ specific isotype plays in these integrin dependent processes warrants further investigation.

A precise balance between cellular adhesion and proliferation is required for cell homeostasis, particularly in the regulation of immunity. Retinoids are well established in their ability to regulate proliferation in a variety of cell types, and their role in modulating cellular adhesion is becoming more clear. Our results shown in Fig 4A are consistent with a previous study in K562 cells utilizing the RAR gamma agonist, CD437 [45]. Further, this same study reports that within 24 hours CD437 increases expression of p21, a cyclin-dependent kinase inhibitor. [45]. Interestingly, it has been demonstrated that overexpression of the beta 1 integrin subunit results in a marked increase in the expression level of p21 [46]. A number of studies have suggested that p21 is capable of inducing apoptosis (for review see [47]). Hsu et al report that apoptosis was observed in K562 cells after 144 hours of exposure to CD 437 [45]. It is plausible to suggest that the RARγ agonist, CD 437, may be increasing expression of both p21 and the beta 1 integrin and synergistically they may play a role in cellular growth arrest and potentially apoptosis at later exposure times.

The current study provides insight into the role of RAR specific isotypes in K562 cellular adhesion and proliferation. Our work is the first to demonstrate that treatment with the RARγ specific agonist augments cellular adhesion in a time- and concentration- dependent manner to α5β1 integrin substrates, increases cell surface levels of the β1 integrin subunit, and dampens cellular proliferation in a time and concentration dependent manner in a human erythroleukemia cell line. To shed light on how this particular agonist may synergistically, yet reciprocally regulate cellular adhesion and proliferation, future studies will need to characterize the specific RARγ-RXR partnerships involved in cellular adhesion and proliferation, determine how the α5β1 integrin ligation status effects proliferation in the presence of this agonist, and identify the particular cellular signals responsible for the reciprocal regulation between cellular adhesion and proliferation that is propagated by retinoid treatment. The therapeutic use of retinoic acid is largely limited due to its numerous side effects; however, retinoids through their associated RARs play a pivotal role in controlling immunity. Illuminating the role of each RAR isotype would allow for strategic targeting of these receptors, which would profoundly impact immune regulation.
